# Therapeutic Effect of Oral Bisphosphonates on Choroidal Neovascularization in the Human Eye

**DOI:** 10.1155/2010/206837

**Published:** 2010-07-25

**Authors:** Shigeru Honda, Takayuki Nagai, Naoshi Kondo, Masahide Fukuda, Sentaro Kusuhara, Yasutomo Tsukahara, Akira Negi

**Affiliations:** Department of Surgery, Division of Ophthalmology, Kobe University Graduate School of Medicine, Kobe 650-0017, Japan

## Abstract

*Purpose*. Choroidal neovascularization (CNV) is often associated with age-related macular degeneration (AMD) and pathological myopia (PM). Bisphosphonates, the drug of choice to treat osteoporosis, have been recently reported to have anti-angiogenic effects. The purpose of this study is to investigate the therapeutic effects of oral bisphosphonates for CNV in humans. *Methods*. Thirty-six consecutive cases with CNV due to AMD or PM who declined anti-VEGF therapy were recruited. The patients were prescribed 5 mg of oral alendronates daily for 6 months. The best-corrected visual-acuity (BCVA), the lesion size in fundus photographs and fluorescein angiography, foveal thickness and total macular volume in optical coherence tomography were compared between pre- and post-treatment. *Results*. The mean BCVA of the patients was significantly improved after a months with the treatment in the AMD group. In the PM group, the mean BCVA was maintained up to 6 months with the treatment. The mean lesion size was significantly decreased by 3 months in both groups. The averages of foveal thickness and total macular volume were significantly reduced after 1 month of treatment in the AMD group.*Conclusions*. Oral bisphosphonate should be further investigated as a possible therapeutic and preventive drug for CNV due to AMD and PM.

## 1. Introduction

Choroidal neovascularization (CNV) is a major cause of adult blindness and is often associated with age-related macular degeneration (AMD) and pathological myopia or other macular diseases. Current therapies for CNV are represented by repeated intravitreal injections of antivascular endothelial growth factor (VEGF) antibodies or aptamer [1, 2]. They require periodic reinjections of the antibody or aptamer every 4–6 weeks for many years to keep the lesion stable and to maintain vision, or else visual acuity was reported to be reduced [3]. These retreatments may pose a cumulative risk for ocular and systemic complications such as endophthalmitis and strokes [4–6] and pose a burden on both the patients and health care systems. Hence, we are interested in alternative therapies using oral drugs or eye drops, since they are usually less expensive, easier to use to treat a broad range of patients, and may also be used for prevention [7].

Bisphosphonates are powerful inhibitors of osteoclasts, and are commonly used as the drug of choice to treat and prevent osteoporosis [8]. Recent studies have demonstrated that the antitumor and antiangiogenic effects induced by suppressing VEGF expression are associated with bisphosphonates, which opens up novel possibilities for this drug class and raises new areas of research for tumorigenesis and angiogenesis [9–12]. However, in ophthalmology, bisphosphonates are known only as drugs that may cause uveitis, scleritis, or orbital inflammation as rare side effects [13–15]. Although bisphosphonates were thought to accumulate mostly in the bone tissue, we suspected that bisphosphonates may have good permeability into the eye, even to the point of causing side effects in rare cases. Following these insights, we recently demonstrated the inhibitory effects of bisphosphonates on laser-induced choroidal neovascularization (CNV) due to suppressed VEGF expression in mice, and hypothesized about the therapeutic effects of this drug in human CNV associated with AMD and related diseases [16]. 

Therefore, we conducted a preliminary prospective study, in which we administered oral bisphosphonates to 36 cases of CNV secondary to AMD and pathological myopia, and found that oral bisphosphonates significantly inhibited CNV in these patients.

## 2. Methods

The cohort included 40 eyes from 36 consecutive patients with CNV secondary to AMD and pathological myopia (25 eyes of 24 AMD cases and 15 eyes of 12 pathological myopia cases), who declined intravitreal injections of anti-VEGF drugs or had systemic risks for strokes. Since both types of CNV are known to respond to anti-VEGF therapy [1, 17], we included both phenotypes in the present study. The CNV was determined by slit-lamp biomicroscopes of the fundi, 50 degree color fundus photographs, optical coherence tomographies (OCT) (Stratus or Cirrus OCT, Carl Zeiss Meditec Japan, Tokyo, Japan), fluorescein angiographies (FA) and indocyanine green angiography (ICG). All CNV cases included subfoveal lesions accompanied by serous retinal detachments and subretinal hemorrhages in this study. The AMD cases (25 eyes) included all 3 lesion types, namely, predominantly classic (6 eyes), minimally classic (11 eyes) and occult with no classic (8 eyes), lesions determined by FA. Eleven cases with recurrent CNV once maintained by photodynamic therapy (PDT) from 3 months to two years ago were also included in the AMD cases. Cases of polypoidal choroidal vasculopathy (PCV) were diagnosed by ICG and were excluded from this study since PCV is not thought as typical CNV [34]. Pathological myopia was diagnosed by an axial length over 26.5 mm and the corresponding fundus findings. No patients received prior treatment in the myopia cases. The summarized clinical data of the enrolled patients are shown in Table 1. The visual acuities were determined using a Landolt C chart and were converted to a logarithm of the minimum angle of resolution (LogMAR) for calculation and description. The lesion size was analyzed by NIH image J software using digital images. Specifically, the lesion size was determined as the combined area of dye leakage at 5 minutes in FA and hemorrhage in fundus photography and was quantified by the number of pixels. All subjects were masked for patient ID, clinical course, and the date of examinations.

Under approval by the Kobe University institutional review board and informed consent from all the patients, 5 mg of oral alendronates (Teijin Pharma. Ltd., Tokyo, Japan) were prescribed daily for six months. The patients were informed about the side effects of bisphosphonates (e.g., gastritis, arthralgia, uveitis) and were advised to consult their physician if they felt any unusual symptoms. Medication compliance was checked by interview and ophthalmological examinations, including the best-corrected visual-acuity (BCVA), color fundus photographs, and OCT, which were performed monthly. Fluorescein angiographies were repeated at three months posttreatment.

The LogMAR BCVA was evaluated as the main outcome of the treatment. The change in lesion size found from fundus photographs and FA, the foveal thickness (average of central 1 mm circle), and the total macular volume determined automatically by a 6 mm retinal map from Stratus OCT and by a 200 × 200 retinal map from Cirrus OCT were also assessed over the treatment period. The OCT system used in each patient was consistent throughout the follow-up period.

For statistical analysis, Wilcoxon signed-rank test was used for the BCVA, lesion size, foveal thickness, and total macular volume. *P* values less than.05 were considered to be statistically significant.

## 3. Results

Twenty-one eyes of 20 AMD cases were ultimately followed-up for 6 months, since three cases self-discontinued and one preferred photodynamic therapy after 3 months of medication. Fourteen eyes of 11 pathological myopia cases were followed-up for 6 months, but one case discontinued medication after 3 months because of bilateral uveitis, which resolved with topical steroids. 

**Figure 1:**
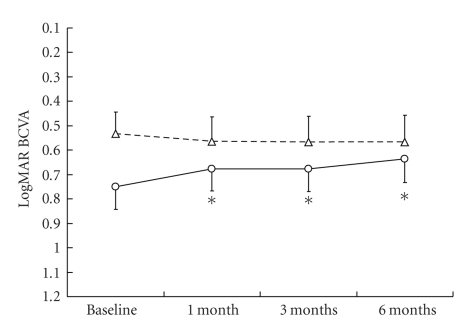
Time-course of the LogMAR BCVA values with oral bisphosphonates. Solid line; AMD, dashed line; myopic CNV. The values are presented as means + SEM. *P < .05. LogMAR: logarithm of the minimum angle of resolution, BCVA: best-corrected visual acuity.

**Figure 2:**
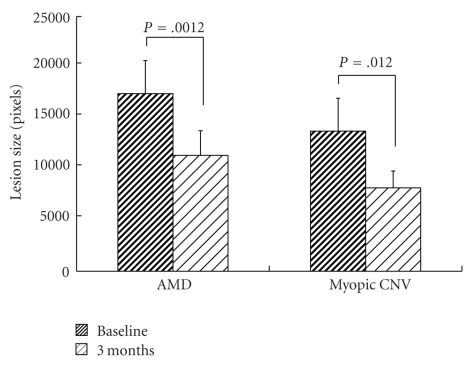
Change in lesion size with oral bisphosphonates. The values are presented as means + SEM.

**Figure 3:**
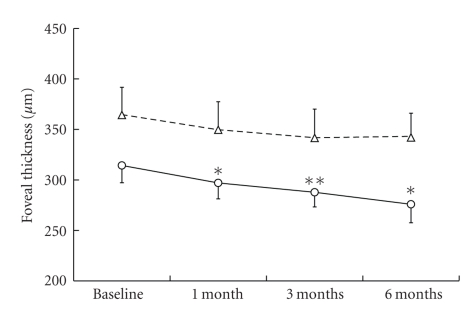
Time-course of the foveal thickness with oral bisphosphonates. Solid line; AMD, dashed line; myopic CNV. The values are presented as means + SEM. *P < .05, **P < .01.

**Figure 4:**
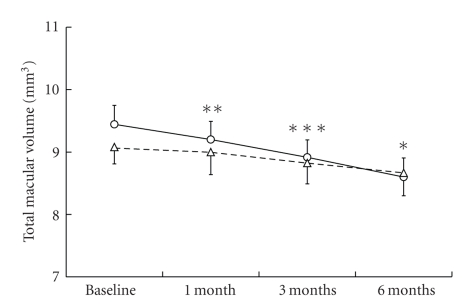
Time-course of the total macular volume with oral bisphosphonates. Solid line; AMD, dashed line; myopic CNV. The values are presented as means + SEM. *P < .01, **P < .005, ***P < .001.

**Figure 5:**
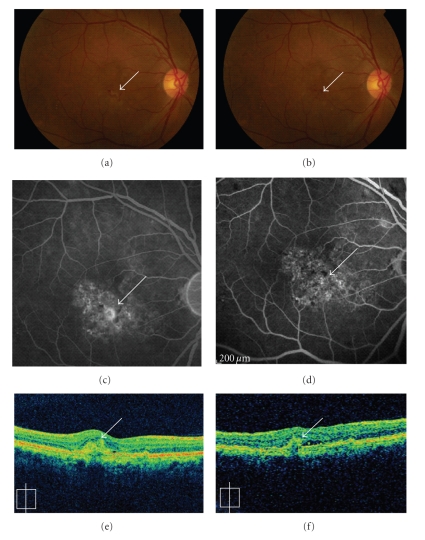
A 61-year-old male showed CNV (arrows) due to AMD (a). Fluorescein angiography (FA) showed predominantly classic CNV (c), and optical coherence tomography (OCT) revealed a subretinal lesion before treatment (e). After the oral administration of alendronate for three months, the CNV regressed remarkably (b) and no leakage was observed using FA (d). A remarkable decrease in the size of the subretinal lesion was observed using OCT (f).

**Figure 6:**
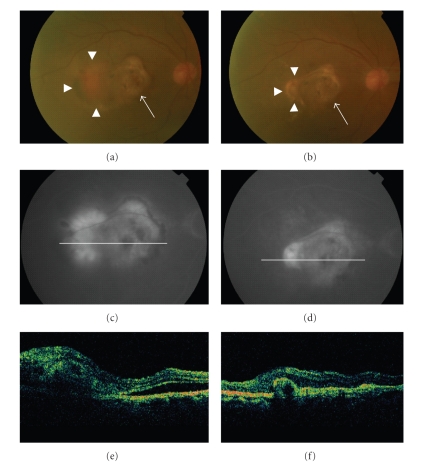
A 79-year-old male who received PDT for 27 months ago showed a recurrence of CNV (arrowheads), mainly at the temporal edge of the old scar (arrows) (a). Fluorescein angiography (FA) showed predominantly classic CNV (c), and optical coherence tomography (OCT) revealed a subretinal lesion before treatment (e). After the oral administration of alendronate for three months, the CNV became subretinal fibrosis (b) and no leakage was observed using FA (d). A remarkable decrease in the size of the subretinal lesion was observed using OCT (f).

**Figure 7:**
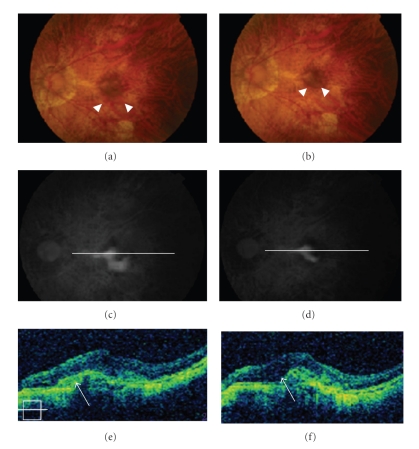
A 57-year-old female was referred for myopic CNV (a). Predominantly classic CNV was detected by FA (c), and a sub-retinal lesion with pigment epithelial detachment was found by OCT (e). With three months of oral alendronate administration, the lesion decreased remarkably in size (b), and the dye leakage was attenuated as detected by FA (d). OCT showed a decrease in the size of the subretinal lesion (arrows) (f).

The mean BCVA of the patients was significantly improved after a month with the treatment in the AMD group (Figure 1). Comparing the proportions of BCVA between pretreatment and after 6 months of the treatment, the number of eyes in which BCVA improved more than 0.2 LogMAR,, changed within 0.2 LogMAR and deteriorated more than 0.2 LogMAR were 5 (24%), 15 (71%), and 1 (5%) eyes, respectively in the AMD group, whereas those in the pathological myopia group were 2 (14%), 9 (64%), and 3 (22%) eyes, respectively. The mean lesion sizes determined in FA and fundus photograph were significantly decreased after 3 months of the treatment in both groups (Figure 2). The mean foveal thickness (Figure 3) and total macular volume (Figure 4) measured by OCT were significantly decreased in the AMD group after a month with the treatment. No adverse systemic side effects were found or self-reported in any of the present cases. The detailed findings from selected cases of the responders are shown in Figures 5, 6, and 7.

## 4. Discussion

In our preliminary study, we found that oral bisphosphonates stopped the progression of CNV due to AMD and pathological myopia in 40 eyes from 36 patients. Their BCVA was maintained for at least 6 months, and the measured lesion size was significantly reduced with the treatment. In the AMD group, OCT revealed a significant improvement in foveal thickness and total macular volume at one month after the treatment, and further improvements were observed up to 6 months with the treatment. 

Although recent studies have demonstrated that antitumor and antiangiogenic effects are associated with bisphosphonates [9–12], in the field of ophthalmology, they are regarded only as drugs that may cause ocular inflammation in certain individuals [13–15]. Therefore, this is the first study which reports beneficial ocular effects of bisphosphonates. CNV due to AMD and pathological myopia is a representative idiopathic angiogenic disorder and a major age-related disorder in addition to osteoporosis, and the number of patients suffering from these pathologies has increased remarkably over recent years [18, 19]. Hence, many elderly people face the risk of both diseases. Therefore, we considered that oral bisphosphonates might be beneficial for both osteoporosis and AMD. The current therapies for CNV accompanied by AMD and pathological myopia consist of repeated intravitreal injections of anti-VEGF antibodies [1, 17]. Anti-VEGF therapy requires monthly re-injections of the antibodies for a long period to maintain vision, which poses a cumulative risk for ocular and systemic complications [4–6]. Moreover, they are not suitable for the prevention of disease. Hence, recent reviews have pointed out the need for an optimal strategy for anti-VEGF therapy, including combinations with other therapies [1, 20, 21].

Autoradiography with an intraperitoneal administration of [^14^C]-alendronate in mice revealed that it accumulates not only into the bone tissue, but also in the eye [22], particularly in the choroidal tissues (personal communication with Teijin Pharma. Ltd.). This may explain the delivery of oral alendronates to CNV lesions. Alendronate is a nitrogen-containing bisphosphonate (N-BP) which inhibits famesyl diphosphonate synthase in the biosynthetic mevalonate pathway. Likewise, statins are known to suppress CNV via an inhibition of mevalonate synthesis [23]. Recent reports have demonstrated that N-BP inhibits the expression of VEGF, matrix metalloproteinase (MMP), and the integrin families to inhibit angiogenesis both *in vitro* and *in vivo* [8–11, 16]. The involvement of MMP and integrins as well as VEGF in CNV formation has been well documented [24]. Alendronate may suppress CNV via a direct inhibition of the proliferation of vascular endothelial cells [25], and by regulating cellular angiogenic gene expression [16, 26, 27]. In addition, bisphosphonates are known to control the inflammation induced by macrophages and mononuclear cells [28–30]. Although AMD is strongly associated with several molecules which have important roles in inflammation [31, 32], a recent report suggested that myopic CNV is not associated with these molecules [33]. This may explain the better response of AMD to bisphosphonates than myopic CNV.

Currently, a number of bisphosphonates have been developed, and some of them are exempt from serious ocular adverse events [34]. The broad beneficial effects of bisphosphonates are being investigated in many research areas besides osteoporosis, especially tumorigenesis. Hence, we considered that the effects of oral bisphosphonates may be further investigated as a therapeutic drug for CNV, including AMD and pathological myopia. Moreover, the combination of oral bisphosphonates with anti-VEGF therapy can be further investigated since it may reduce the number of anti-VEGF therapy visits required.

Due to the small sample size and non-randomized controlled study, a definitive conclusion may not be drawn from these results, and further studies including a randomized controlled trial and comparisons with other therapies such as intravitreal ranibizumab are required to confirm the safety and efficacy of bisphosphonates in the eye. However, it is interesting that our previous study using photodynamic therapy to treat AMD did not improve the mean visual acuity of the patients, which might imply some advantages of oral bisphosphonates for specific AMD patients. Our goal is to determine whether bisphosphonates are useful as preventative or supportive drugs for current anti-VEGF therapy in CNV treatment. The present study gave a novel and totally different insight into the use of this drug class in ophthalmology and suggested a new possibility for the management of CNV accompanied by AMD or pathological myopia.

## 5. Summary

We treated patients with choroidal neovascularization (CNV) due to age-related macular degeneration and pathological myopia with oral bisphosphonates and found their remarkable therapeutic effect on CNV for the first time. Our findings suggest a beneficial effect of bisphosphonates in the treatment, and perhaps the prevention, of CNV for the future.

## Figures and Tables

**Table 1:** Clinical data of CNV patients treated by oral bisphosphonate.

CNV type	AMD	Pathological myopia
Age (years)	76.0 ± 9.0	64.4 ± 8.7
Gender (male/female)	20/4	2/10
Baseline LogMAR BCVA	0.75 ± 0.46	0.53 ± 0.34

BCVA: best corrected visual acuity, LogMAR: logarithm of the minimum angle of resolution.
